# Evidence for a general cognitive structure in pigeons (*Columba livia*)

**DOI:** 10.1007/s10071-024-01912-3

**Published:** 2024-11-02

**Authors:** Mary Flaim, Aaron P. Blaisdell

**Affiliations:** 1 Ruhr-Universität Bochum, Bochum, Germany; 2 University of California – Los Angeles, Los Angeles, United States

**Keywords:** Intelligence, Test battery, Pigeon, G factor

## Abstract

A well replicated result in humans is that performance, whether good or bad, is consistent across a wide variety of cognitive tasks. Factor analysis extracts one factor that can account for approximately half of the variance in performance. This factor is termed *g* and almost all cognitive tasks positively load onto this factor. While some neurobiological correlates of *g* have been identified in humans, causal experiments are only feasible in animals. When mice and some avian species are assessed with cognitive test batteries, performance positively correlates, and the first component extracted has similar properties to *g*. There are some limitations to the species tested thus far, including comparability in the cognitive domains assessed. The pigeon is an ideal subject to overcome these issues since pigeons, humans, and other primates are frequently given similar tasks and many neural correlates of performance have been identified in the pigeon. We created a test battery that assessed different domains, including associative learning, memory, cognitive flexibility, and reaction time. When all tasks were included, there was evidence for a two-component structure that was influenced by subjects’ age. When the reaction time task was excluded, there was a *g-like* component. The implications for these results when constructing future test batteries and comparing across species are discussed.

## Introduction

When a person performs well on one cognitive task, they are likely to perform well in another, even if the tasks assess different domains of cognition (Carroll 1993; Deary 2000; Spearman 1904). This effect has been replicated many times, but the most compelling results are from full scale intelligence quotient (FSIQ) tests because of the number and variety of tasks used. The exact number and type included vary from test to test, but they typically include 11–17 measures that assess memory, basic math, spatial reasoning, and analogical reasoning (Carroll 1993; Johnson et al. 2004). Despite differences in the test batteries and variety of tasks used, a positive correlation matrix is found (Carroll 1993; Johnson et al. 2004). When variable-reducing techniques, like principal component analysis (PCA) or factor analysis, are applied to this positive correlation matrix, one factor is consistently extracted that can account for approximately half of the variance (Carroll 1993; Deary 2000). All cognitive tasks positively load onto this factor and because this factor is seemingly related to all cognitive abilities, it is referred to as *g* for “general” (Carroll 1993; Deary 2000; Spearman 1904). *g* is one of the most well-replicated results in psychology and there are important parameters to consider when extracting this factor. The tasks used for assessment and the size and homogeneity of the sample population being assessed can affect the quality of the factor extracted, which has been reviewed elsewhere (Bonett and Wright 2000; Carroll 1993; Deary 2000; Gignac 2015; Jensen 1992; Johnson et al. 2004; Warne and Burningham 2019). Briefly, to extract the strongest *g* factor, a large, heterogeneous sample of people should be given a large variety of cognitive tasks, that are reliable and sensitive to individual differences (Bonett and Wright 2000; Carroll 1993; Deary 2000; Gignac 2015; Jensen 1992; Johnson et al. 2004; Warne and Burningham 2019).

Even though *g* is well replicated, there are two main theories for what *g* is. One theory argues that *g* is composed of more specific cognitive processes like working memory (WM), short-term memory (STM), processing speed, attention, and associative learning (Conway et al. 2002; Deary 2000; Jensen 1998; Kaufman et al. 2009; Sheppard and Vernon 2008). It is likely that the tasks included in FSIQ tests, particularly complex tasks that load highly onto *g*, require support from multiple cognitive domains (Chuderski 2013). Therefore, *g* could reflect individual differences in how many processes are required to solve a task (Chuderski 2013). Another theory suggests that differences in one of these abilities could act as a bottleneck, constraining and weakening the ability of all other cognitive domains to function (Kovacs and Conway 2019). With this theory, *g* primarily reflects differences in one cognitive ability, but it is unclear which. These theories are helpful for understanding the more specific cognitive processes that are involved with intelligence tests and how those processes are used across a large number of tasks (Conway et al. 2002; Deary 2000; Jensen 1998; Kaufman et al. 2009; Sheppard and Vernon 2008). Future research is still needed, however, to fully understand if there is a relationship between these cognitive processes that could impact the positive correlation matrix (Frischkorn et al. 2019).

At the psychological construct level, *g* is related to a variety of cognitive processes. Similarly, *g* is correlated with a variety of neurobiological mechanisms, processes, and features (Deary et al. 2010). A variety of methods, including functional magnetic resonance imaging (fMRI), positron emission tomography (PET), and lesions due to accident or stroke, have indicated the importance of the frontal cortex in a wide variety of tasks (Jung and Haier 2007). The dorsolateral prefrontal cortex (dlPFC) in particular is active during a variety of WM and reasoning tasks, though similar patterns of activations in different areas of the frontal cortex for other types of tasks have also been identified (Colom et al. 2013). Yet, brain areas do not function in isolation; rather, different areas are connected, forming functional networks (van den Heuvel & Sporns, 2013). Similar performance across tasks may be partially due to how whole networks are activated by tasks instead of discrete regions. A network connecting the frontal and parietal cortex is implicated (Fraenz et al. 2021; Jung and Haier 2007; Zanto and Gazzaley 2013). Thus, most research has indicated that consistent performance could be due to activation of the frontal cortex over a wide variety of tasks (Colom et al. 2013; Jung and Haier 2007).

Since the neurological correlates indicate that a wide variety of cognitive tasks depend on the PFC, it is possible that animals with an equivalent region would also show a *g* like factor. Investigations over the past 20 years have generated promising results that are described in more detail elsewhere, but investigations with a wide variety of species have generally been successful (Burkart et al. 2017; Flaim and Blaisdell 2020; Shaw and Schmelz 2017). For mice, Matzel and colleagues in particular have been consistently exploring a general factor using a cognitive test battery of 5 tasks that target different domains of learning (Matzel et al. 2003; but also see Locurto and Scanlon 1998; Locurto et al. 2003, 2006). Multiple experiments found that performance was positively correlated across all tasks, and the first factor extracted could account for 38–43% of the variance in performance (Kolata et al. 2005, 2007, 2008; Matzel et al. 2003, 2006). Subsequent experiments have shown that performance on this cognitive test battery is positively correlated with measures of WM, similar to what is seen in humans (Kolata et al. 2005). For most avian species tested thus far, similar results to mice and humans have been found, with the exception of song sparrows (Anderson et al. 2017; Boogert et al. 2011). For song sparrows, two factors were extracted, and not all tasks loaded onto the first factor extracted. This may be due to the low reliability in performance across years on cognitive tasks in song sparrows (Soha et al. 2019). While the results from animals thus far are interesting and promising, there are some difficulties in comparing *g* across species.

Research with many species thus far indicates that *g* can be found beyond humans, but it is not clear exactly how similar *g* is across species. This ambiguity is partially due to the differences in test batteries. In humans, the time it takes to perform very simple cognitive tasks shows a very consistent, though weak relationship to *g*, where more intelligent individuals are consistently faster (Sheppard and Vernon 2008), yet this has not been investigated or replicated with animal test batteries (see discussion by Flaim and Blaisdell 2020). In contrast, the relationship between response inhibition and *g* has rarely been investigated in humans, but response inhibition tasks are almost always included in avian cognitive test batteries (Flaim and Blaisdell 2020). Even when the cognitive domain does overlap, there are differences in the procedures used for humans versus nonhumans that can impede comparisons. Taking associative learning as an example, in humans an initial investigation using a simple associative learning task, where children had to learn which picture was associated with a reward, was not related to IQ scores (Plenderleith 1956). More recent investigations have used the word-pairs task, where participants learn up to ten arbitrary pairs of words, like cat-pie, or the three-term contingency task where one word serves as a cue and the participant must learn three response words (Kaufman et al. 2009). These associative learning tasks show a positive relationship to *g* that scales with complexity, where the more complex three-term contingency task has a stronger relationship with *g* (Tamez et al. 2008; Williams and Pearlberg 2006; but see Kaufman et al. 2009). In contrast, for mice and birds, a simple associative learning task, such as learning how to discriminate one cue from another to obtain a food reward, is related to the *g* like factor extracted in these species (Flaim and Blaisdell 2020). The finding that associative learning is related to *g* across species, but different levels of difficulty are needed to reveal such a relationship, may be related to the experience of the subject. *g* is related to complexity, but it is also related to novelty, where novel tasks tend to have a high *g* loading (Carroll 1993; Sternberg and Gastel 1989). If animal subjects are naïve to any highly artificial experimental stimuli and procedures, the task may be sufficiently novel to explain why performance is related to *g*, despite the apparent simplicity. This lack of experience could also make the task more difficult for animals. While experimenters tend to see the associative learning tasks given to animals as ‘simple’, it has been argued that these tasks rely on learning which cues in the environment are the most informative (Pearce and Bouton 2001; Rescorla, 1988). Historically, attention has been highlighted as an important mechanism in this learning process, but more recently it has been emphasized that detecting the consistency between two events could form the basis for propositional relationships or causal reasoning (De Houwer and Hughes, 2016; Pearce and Bouton 2001). This is a relatively demanding series of cognitive processes that could become easier with repeated practice (De Houwer & Hughes, 2016). For instance, when many humans are assessed, they have had years of experience in an educational setting with similar materials and task demands as the word-pairs and three-term contingency tasks. Therefore, for humans, task difficulty may be a more important factor for investigating associative learning and *g*. This example highlights the relative importance of novelty and complexity when comparing test batteries and the extracted *g* factor across species.

While animals may be relatively naïve to cognitive assessments compared to most human samples, there are other issues when comparing across species. In nonhuman research on *g*, the sample of animals tested is often homogeneous in some way (Shaw and Schmelz 2017). Mouse studies typically use males reared in standard laboratory conditions (Kolata et al. 2008; but see Galsworthy et al. 2005; Sauce et al. 2018). For testing avian subjects, like robins, in the wild, collection could be biased towards bolder or more exploratory individuals (Shaw and Schmelz 2017). If *g* is a robust phenomenon in animals, then it should replicate across members of the species that differ from the samples assessed thus far, for example in sex, personality, or environmental conditions. In addition, most experiments assess a small number of subjects (Bonett and Wright 2000; Mundfrom et al. 2005; but see de Winter et al., 2009). This can be overcome by using a consistent test battery, which makes it possible to pool results from multiple experiments, as demonstrated by Kolata et al. (2008). Utilizing a species that is more commonly investigated, either in the lab or across field sites, could also increase the number of subjects if multiple labs are willing to work together (Shaw and Schmelz 2017). Thus, there could be improvements in both the test battery and sample characteristics, particularly for avian species. Tasks that assess clear cognitive domains, facilitate cross species comparisons, and have identified neural correlates should be favored. Species for which it is possible to obtain a large and diverse sample should also be favored, at least in these preliminary investigations of *g* in animals.

Given these arguments, it is surprising that pigeons have not been given a comprehensive test battery, given their long history as an animal model in psychology. Pigeons have excellent visual acuity and readily learn to peck visual stimuli in a touchscreen operant chamber, similar to procedures used to assess human and nonhuman primates (Wright et al. 2018; Zentall 2021). Investigations of matching, interval timing, reaction time (RT), memory, and many other cognitive domains show there are similarities in performance across pigeons and primates that indicate similar underlying mechanisms at the psychological and neurobiological level (Colombo and Scarf 2020; Güntürkün 2005; Vickrey and Neuringer 2000; Zentall 2021). Methods for investigating memory, associative learning, and cognitive flexibility in particular have been well established, and the neural mechanisms supporting performance have been identified on some level. Similar to humans, performance on many cognitive tasks seems to depend on nidopallium caudolaterale (NCL) which is the avian equivalent to the mammalian PFC (Güntürkün 2005). For example, when assessing STM in the pigeon by requiring pigeons to remember a stimulus over a short delay to guide choice behavior (note that performance can also be affected by interference, so this is not necessarily a pure measure of STM), there is sustained neural activity in the NCL that relies on the neurotransmitter dopamine, similar to results found in nonhuman primates (Johnston et al. 2017; Karakuyu et al. 2007). Given this rich history, there are many tasks that could be included in a cognitive test battery for pigeons, but a few were selected for our initial investigation.

The tasks in the battery developed here were selected according to how well they assessed a specific cognitive domain, if the task facilitates cross-species comparisons, and if the neural substrates of performance had been identified (Diekamp et al. 2000; Flaim and Blaisdell 2020; Izquierdo et al. 2017; Johnston et al. 2017; Karakuyu et al. 2007; Lissek et al. 2002; Vickrey and Neuringer 2000). Ultimately, the pigeon cognitive test battery was designed to assess associative learning, cognitive flexibility, memory, and RT. Specifically, there were four tasks, symbolic match to sample (SMTS), serial reversal learning, delayed match to sample (DMTS), and a choice RT task. All the tasks were sufficiently sensitive to detect individual differences in performance, and all subjects completed at least two tasks in the battery (Table 1). For two tasks, the DMTS and RT task, the middle and end of the total training given seemed suitable for capturing cognitive differences across subjects and both time points were investigated (Locurto et al. 2006). While the choice RT task we developed was similar to the Hick’s RT task used for humans, the resulting measure for pigeons differs in a potentially critical way. In humans, it is theoretically possible to separate how long it takes participants to make a decision (‘decision time’ or DT) and how long it takes them to complete the movement (‘movement time’ or MT; Jensen 1982). The relationship between DT and *g* is very consistent, while MT is more variable (Longstreth 1984; Nettelbeck, 1998). Sometimes researchers find similar correlations between MT and *g* as between DT and *g* (Carlson and Jensen 1982; Stough et al. 1995), whereas others find MT is not related to *g* at all (Beauducel and Brocke 1993; Buckhalt et al., 1998). It was not possible to separate the RTs from pigeons into DT and MT, thus it was not clear how well this task captures a cognitive component that would theoretically be related to *g*. Thus, we conduct two PCAs, one with and one without inclusion of the RT task.

The correlation matrices were not uniformly positive and seemed to form two clusters, with the SMTS and RT tasks forming one cluster and the DMTS and reversal learning tasks forming another. When the RT task was included in the PCA, two components were extracted, and neither component had uniformly positive loadings. When the RT task was excluded, all tasks positively loaded onto the first component extracted. Overall, upon closer examination of the tasks, principal components, and the influence of the subjects’ age, there was more evidence for a factor similar to *g*. The differences in the task loadings onto the first component, subject age, and potential procedural issues are discussed.

**Table 1:** All subjects in the test battery and which tasks they completed

Subject Information				Tasks and Dependent Measures							
Name	Age		Other Tasks	SMTS	RT - Mid	RT - End	Reversal Learning	DMTS Int - Mid	DMTS Slope - Mid	DMTS Int - End	DMTS Slope - End
Athena		1	0	18	0,82	0,82	0,55	0,36	0,03	0,98	-0,14
Wenchang		1	0	14	0,78	0,69	0,75	1,10	-0,19	1,45	-0,10
Itzamna		2	2		0,73	0,78	0,67				
Odin		2	1		1,35	0,97	0,73				
Luigi		4	0	31	0,68	0,57	0,38	0,47	-0,90	1,03	-0,47
Shy guy		4	0	17	0,87	0,79	0,64	0,85	-0,18	1,43	-0,51
Bowser		4	2	10	0,59	0,59	0,52	0,93	-0,37	1,25	-0,11
Wario		4	0	8	1,29	1,00	0,31	1,02	-0,52	0,98	-0,31
Waluigi		4	1	19	0,92	0,87	0,27	1,05	-0,44	1,68	-0,23
Peach		4	1	15	0,75	0,77	0,7	1,06	-0,13	1,20	-0,14
Mario		4	0	25	1,12	1,22	0,62	1,49	-0,16	1,35	-0,15
Herriot		12	7	17	0,66	0,69	0,52	0,74	-8,46	1,14	-0,86
Goodall		12	6	9	0,82	0,73	0,36	0,79	-0,18	0,83	-0,17
Durrell		13	6		0,73	0,83		1,08	-0,33	0,96	-0,25
Darwin		13	11		0,94	1,05	0,6	1,41	-0,11	1,42	-0,04
Cousteau		13	5	16	1,45	1,76	0,12				
Jubilee		17	9	35	0,95	1,00	0,34	0,73	-0,95	0,55	-0,38
Gambit		17	9	14			0,74	1,11	-0,20	1,45	-0,20
Estelle		18	6	30	0,91	1,19	0,44	1,14	-0,08	1,38	-0,09
Dickinson		18	9	35	2,12	2,11	0,55	1,30	-0,22	1,39	-0,13
Vonnegut		18	10	21	1,08	0,90	0,62	1,68	-0,56	1,30	-0,13
Hawthorne		18	9	35	1,09	1,31	0,24				

Other tasks refers to the number of cognitive tasks completed before or between tasks in the cognitive test battery and age is how old each subject was in years. SMTS is symbolic match to sample, RT is reaction time, and DMTS is delayed match to sample. The values inside of the table correspond to the dependent measures used to assess performance. For the RT and DMTS tasks, two time point of training were investigated, the relative middle (mid) and end points of training for each task. For each time point, performance was assessed by taking an aggregate across three sessions. For the DMTS task, ‘int’ stands for intercept

## Method

### Subjects

Twenty-two pigeons served as subjects. The age at the start of the test battery ranged from 1 to 18 years old and ten were female. Subjects varied in how much experience they had with other cognitive tasks (Table 1). Subjects were individually housed in steel home cages with metal wire mesh floors in a vivarium. They were maintained at 80% of their free-feeding weight and provided free access to water and grit while in their home cages. Testing occurred at approximately the midpoint of the light portion of the 12-hour light-dark cycle. All procedures were approved by the UCLA Institutional Review Board.

### Apparatus

Testing was conducted in a flat-black Plexiglas chamber (38 cm wide x 36 cm deep x 38 cm high). All stimuli were presented by computer on a color LCD monitor (NEC MultiSync LCD1550M) visible through a 23.2 × 30.5 cm viewing window in the middle of the front panel of the chamber. The bottom edge of the viewing window is 13 cm above the chamber floor. Pecks to the monitor were detected by an infrared touchscreen (Carroll Touch, Elotouch Systems, Fremont, CA) mounted on the front panel. A custom-built food hopper (Pololu, Robotics and Electronics, Las Vegas, NV) was located in the center of the front panel, its access hole flush with the floor. The food hopper contained a mixture of leach grain pigeon pellets and seed (Leach Grain and Milling). All experimental events were controlled and recorded with a variety of personal computers running the Windows 10 operating system. Stimuli were presented using the 2.7.11 version of Python with the psychopy toolbox, version 3.0.3 (Peirce 2007).

### Procedure

The tasks are described briefly here to emphasize the features that are relevant to the dependent measures ultimately included in the battery, but additional performance data and figures are available at https://github.com/AaronBlaisdell/IntelligentPigeon. Subjects did not receive the test battery in the same order, and it was not possible to fully counterbalance for order effects. Additionally, the time between tasks was not consistent across subjects. Subjects received one session per day, 3–7 days a week. All tasks were appetitive and used 3-s of access to a mixture of grain and seed as a reward.

#### Symbolic match to sample

The SMTS was a measure of associative learning and was conceptually based on the arbitrary word-pairs task given to humans (Kaufman et al. 2009). The task is described in more detail in Flaim and Blaisdell (2023), but will be described briefly here. Instead of words, 18 subjects were presented with pictures of foods and animals which were associated through reinforcement. Subjects were trained on four pairs of pictures, where one of the pictures was always a food item while the other picture was always an animal, obtained from the food-pics database (Blechert et al. 2014). To associate pairs of pictures, trials had two phases, a sample phase and a choice phase. In the sample phase, one picture was shown in the center of the screen and is referred to as the sample. When subjects completed an observing response to the sample, pecking the picture ten times (FR10), the choice phase began. In the choice phase, the sample remained on the screen, and two comparison stimuli were presented below the sample on the left and right of the screen. If subjects pecked the correct comparison once (FR1), they received a food reward. If they pecked the incorrect comparison, a correction procedure was used where the trial repeated starting at the sample phase. Correction trials were not included in the analysis. The correct comparison was presented equally often on the left and right side of the screen and equally often with the three other incorrect comparison stimuli. Subjects were trained with this procedure until they were 80% accurate on each pair in a single session or until they had trained for 35 sessions. The number of sessions to reach criterion was used to measure associative learning ability (Table 1).

#### Serial reversal learning

The serial reversal learning task was used to assess cognitive flexibility, being able to update behavior to reflect changes in environment (Izquierdo et al. 2017). Twenty-two subjects were trained with two stimuli, a blue or yellow circle, presented on the left and right side of the screen. One of the stimuli was always followed by a food reward (S+), while the other was not (S-). Subjects had to make three successive pecks (FR3) to indicate their choice and end the trial. When subjects were selecting the reinforced stimulus with 90% accuracy on two consecutive sessions, on the next session the contingency was reversed. The stimulus that was the S- was now the S + and vice versa. Each session was 50 trials. Subjects were trained on five reversals and performance on the first session of each reversal was used to measure cognitive flexibility (Table 1). Due to computer errors, performance on the first, second, and fifth reversal was only measured for 21 subjects.

#### Delayed match to sample

The DMTS task was used to assess memory, or more specifically the ability to maintain a memory of a stimulus over a short delay (Kangas et al. 2011). The task is described in more detail in Flaim and Blaisdell (2023), but will be described here briefly. Eighteen subjects were trained using a procedure similar to the SMTS task. There are three key differences between the DMTS and the SMTS task, the size of the stimulus set, what determined the correct comparison, and the delay period. Subjects were trained with two stimuli, a red circle and a green circle. First subjects were trained without a delay so the rule determining which comparison was correct could be learned. Similar to the SMTS, during this initial training each trial had two phases, a sample phase and a choice phase. During the sample phase, one stimulus was presented in the center location. After subjects completed the observing response (FR10) to the sample, the choice phase began and the two comparison stimuli were presented. One of the comparisons matched the sample, while the other did not. If subjects pecked (FR1) the matching comparison, they received a food reward. If they pecked the nonmatching comparison, a correction procedure was used where the trial repeated starting at the sample phase. The correction trials were not included in the analysis. Trials were separated by a 13-s ITI with a different background color from the trial, which was consistent throughout the experiment. Each stimulus served as the sample an equal number of times and the correct comparison was presented equally often on the left and right side of the screen. When subjects reached criterion on the initial training, 80% correct on two consecutive sessions, they started the DMTS.

In the DMTS, each trial had three phases, sample, delay, and choice. The sample phase is the same as the training described above. Once subjects had completed the observing response, the sample was removed from the screen, and the delay phase began. The delay could be 0, 2, 4, or 8 s, and each delay length was presented an equal number of times with each combination of correct comparison stimulus and location. After the delay had elapsed, only the comparison stimuli were presented. Again, if subjects pecked the matching comparison, they received a food reward. If subjects pecked the nonmatching comparison, the trial ended without reinforcement. The correction procedure was not used during the DMTS. Subjects were trained on this procedure for 30 sessions. The total amount of training subjects received was determined in advance based on the results from Kangas et al. (2011). To assess performance, the response was log transformed to remove response bias, then fitted to an exponential function (White, 2001). Performance was assessed at the middle and end point of training.

#### Reaction time task

**Fig. 1:**
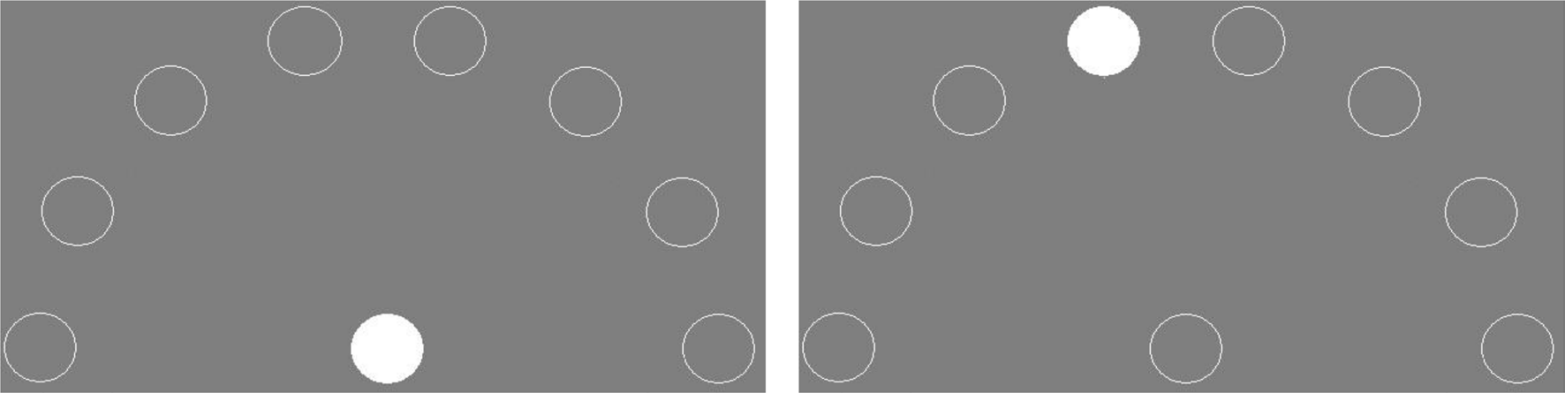
An example trial from the choice RT experiment, where the left image shows the home key phase and the right image shows the choice phase. These images were originally used as a figure in Flaim et al. 2023

This task was used to assess response speed with a procedure that relied on detecting a change in the stimulus display (Sheppard and Vernon 2008). Twenty-one subjects were trained and took part in four different experiments with slight procedural differences overall that are described in more detail in Flaim et al. (2023; *n* = 4–6 for each experiment). The stimulus display consisted of 2–9 circular stimuli that could either be a white outline or completely filled with white. One of the stimuli was in the center of the screen, near the bottom of the viewing window. This stimulus was the home key. The remaining stimuli were arranged in a semi-circle around the home key and were potential targets (PTs). While all subjects were trained with different amounts of PTs present (1, 2, 4, and 8), only the trials with eight PTs have the same procedure across experiments and will be the only condition used in the cognitive test battery. Trials had two phases, a home key phase and a choice phase. During the home key phase, the home key was filled with white and subjects had to peck the home key three times on average (VR3 +/- 2). When subjects completed the peck requirement to the home key, the choice phase began. During the choice phase, the home key became a white outline and one of the PTs was filled with white and became the target (Fig. 1).

If subjects pecked (FR1) the target, they received a food reward. If subjects pecked a PT it was counted as an error of commission, but if subjects did not peck the target or a PT within 5 s, it was counted as an error of omission. Only trials where subjects successfully pecked the target were included in analysis (error rates were very low). The target appeared in each location an equal number of times. For Experiments 1 (*n* = 4) and 2 (*n* = 6) each session had a total of 80 trials and 20 of those trials had eight PTs. Subjects trained for 10 sessions for a total of 200 trials with eight PTs. For Experiments 3 (*n* = 6) and 4 (*n* = 6) each session had a total of 96 trials and 24 of those trials had eight PTs. Subjects were trained for 9 sessions, for a total of 216 trials with eight PTs. Performance was assessed by averaging the median RT from the middle three or last three sessions of the task. The number of trials included in the analysis for each subject ranged from 184 to 216 depending on how many trials had to be excluded due to errors.

### Data analysis

For the SMTS and RT tasks, the data were reverse coded, so a larger number always indicates better performance. To assess reliability in the serial reversal learning task, first a Spearman correlation was used to explore which reversals were most likely to show similar performance and then an intraclass correlation coefficient (ICC) was used to verify those results. To assess reliability for the RT time task, an ICC was used to compare performance at the beginning (first three sessions) and end (last three sessions) of training. For the RT task, because subjects experienced different procedures, an ANOVA was also used to determine if there were any significant differences in median RT across the experiments.

A Spearman correlation with a modified Bonferroni correction was used to compare performance across tasks (Keppel 1982). Age and experience were included as variables that were related to performance (Table 1). PCAs using a Spearman correlation with an unrotated factor solution were used to determine if variance in performance in the test battery could be accounted for with a single component. The ICCs were calculated with SPSS version 28. Since reliability was assessed using a test-retest method, analyses were conducted based on a mean rating, absolute -agreement, 2-way mixed-effects model, with a 95% confidence interval (Koo & Li, 2016). The ‘fit’ function in Matlab version R2023a was used to fit the DMTS to an exponential function. All other statistical analyses were performed using RStudio version 2022.12.0 (R Core Team 2021), with packages readxl (Wickham and Bryan 2023), tidyverse (Wickham et al. 2019), psych (Revelle & Revelle, 2015), ggpubr (Kassambara 2023). The Matlab and R code for data processing and analyses are available at https://github.com/AaronBlaisdell/IntelligentPigeon.

## Results

### Individual cognitive tasks

#### Symbolic Match to Sample

The number of sessions needed to reach criterion, 80% accuracy on all four pairs in a single session, was used as the dependent measure. If subjects did not reach criterion, the maximum number of sessions, 35, was used. The number of sessions required ranged from 8 to 35 (*M* = 20.5, *SD* = 9.13, Table 1). The data were reverse coded, so a larger number indicated better performance.

#### Serial reversal learning

Performance on the first session of the initial discrimination and each reversal was used as the dependent measure. Performance on the initial discrimination and the first and second reversals had weak and nonsignificant correlations with all measures. Performance on the third, fourth, and fifth reversals were significantly, positively correlated with each other (Table 2). This pattern was confirmed where the ICC between the third, fourth, and fifth reversals was 0.822 (CI = 0.639 − 0.92, *p* < .001). This indicated that the initial discrimination and the first and second reversals were not assessing the same ability or cognitive domain as the third, fourth, and fifth reversals, which is also supported by previous research (Izquierdo et al. 2017). Due to the strong correlations between performance on the fourth and fifth reversals, an aggregate measure of performance was created by taking the average across the two conditions. While performance on the third and fifth reversals also had a strong correlation, this may have been due to both conditions having the same reinforced stimulus.

**Table 2:** Correlation matrix between the measures of the serial reversal learning task

Reversal Learning						
		Initial	First	Second	Third	Fourth
First	*rho*	-0.01 (20)	--			
	*p*	0.703				
Second	*rho*	-0.077 (22)	0.164 (21)	--		
	*p*	0.734	0.476			
Third	*rho*	-0.047 (22)	0.045 (21)	0.055 (23)	--	
	*p*	0.837	0.848	0.804		
Fourth	*rho*	0.198 (22)	-0.053 (21)	-0.007 (23)	**0.597 (23)**	--
	*p*	0.376	0.818	0.975	**0.003**	
Fifth	*rho*	0.004 (21)	-0.113 (20)	0.047 (22)	**0.744 (22)**	**0.639 (22)**
	*p*	0.985	0.635	0.834	**< 0.001**	**0.001**

The number inside the parenthesis is the sample size. Bolded values indicate the result was significant after a modified Bonferroni correction (α = 0.04)

#### Delayed match to sample

Performance from the middle (14,15, and 16) or last three sessions (28, 29, and 30) of training was used as the dependent measure. For one subject, Goodall, the 27, 28, and 29th sessions were used due to a computer error in the 30th session. To assess performance, first the data was log-transformed to remove the upper bounds of accuracy and to better estimate discriminability independent of response biases (Kangas et al. 2011). Then the transformed data from the three sessions was fitted to an exponential function for each subject. The intercept of the function indicates the discriminability while the slope indicates the decay of the stimulus over the delay period (White, 2001). The fit for the exponential function was conducted separately for the middle and last three sessions.

#### Reaction time

While explained in more detail in Flaim et al. (2023), justification for the dependent variable used will briefly be given here. Only trials where subjects correctly pecked the target were used in the analysis, because errors were infrequent and difficult to interpret. Median reaction time (RT) from correct trials was used as the dependent measure. To investigate potential effects of task experience, the mean median RT from the first and last 3 sessions of training were compared. An ICC between the mean median RT of the first and last 3 sessions was significant (ICC = 0.945, CI = 0.898 − 0.974, *p* < .001), indicating that performance was reliable across sessions. A 4 × 2 mixed ANOVA, with experiment as a between-subjects factor and task experience as the within-subject factor confirmed there were no statistically significant differences in mean median RT based on which experiment a subject experienced (*F* < 1) or task experience (*F* < 1) and there was no statistically significant interaction between these two factors (*F* < 1). To make the dependent measures more comparable across tasks, the average median RT from the middle (sessions 4, 5, and 6 or 5, 6, and 7) and end point of training (sessions 7,8 and 9 or sessions 8, 9, and 10) were used instead of collapsing across the entire session.

### Cognitive test battery

#### Correlation matrix

A Spearman correlation was used to determine if there was any relationship in performance across the different cognitive tasks. Before the analysis, the data from the SMTS and RT tasks were reversed coded so a larger number indicated better performance for all tasks. For the DMTS and RT tasks, there were two time points during training that could adequately capture individual differences in performance, the middle or end of the predetermined training period. Each time point was analyzed separately in related to the SMTS and serial reversal learning tasks to determine if task experience impacted the relationship between tasks. Age and experience were also included in the analysis since they could have a potential relationship with performance.

The correlation matrix with measures from the middle point of training was not uniformly positive across the cognitive tasks (Table 3). Performance on the SMTS and RT were positively correlated with each other above 0.2, but had correlations closer to zero with the other tasks. Similarly, performance on the DMTS and reversal learning tasks were positively correlated, but had correlations closer to zero with the other tasks. There was also a negative correlation between the RT and intercept measure from the DMTS task. None of these correlations survived a modified Bonferroni correction (α = 0.036, Keppel 1982).

Age and experience had a strong, significant positive correlation with each other and thus had a similar relationship with the cognitive tasks. Age and experience were negatively correlated with almost all cognitive tasks. Only the intercept measure from the DMTS had positive correlation with age and experience.

**Table 3:** Correlation matrix between the measures of the cognitive test battery, age, and experience

Spearman’s Correlations - Middle Point of Training							
Variable		SMTS	RT	DMTS Intercept	DMTS Slope	Reversal Learning	Experience
SMTS	Spearman’s rho	—					
	*p*	—					
RT	Spearman’s rho	0.341 (17)	—				
	*p*	0.181	—				
DMTS Intercept	Spearman’s rho	-0.109 (16)	-0.493 (17)	—			
	*p*	0.688	0.044	—			
DMTS Slope	Spearman’s rho	0.149 (16)	-0.087 (17)	0.265 (18)	—		
	*p*	0.582	0.740	0.286	—		
Reversal Learning	Spearman’s rho	0.195 (18)	0.217 (20)	0.443 (17)	0.386 (17)	—	
	*p*	0.437	0.358	0.075	0.126	—	
Experience	Spearman’s rho	-0.324 (18)	-0.177 (21)	0.387 (18)	-0.172 (18)	-0.137 (21)	—
	*p*	0.189	0.443	0.113	0.495	0.554	—
Age	Spearman’s rho	-0.466 (18)	-0.347 (21)	0.453 (18)	-0.167 (18)	-0.371 (21)	**0.830 (22)**
	*p*	0.051	0.123	0.059	0.509	0.097	**< 0.001**

An aggregate measure was used for the reversal learning task. The number inside the parenthesis is the sample size. Bolded values indicate the result was significant after correcting for multiple comparisons (α = 0.036)

Similar results were obtained when using the measures of performance from the end point of training (Table 4). There was a positive correlation between the SMTS and RT tasks and positive correlations between the DMTS measures and reversal learning task. The correlation between the RT task and the intercept from the DMTS task was still negative, but closer to zero. Like the previous correlation matrix, age and experience were negatively correlated with almost all tasks. For the RT task, the negative correlation was stronger when the end time point of training was used, but for the intercept of the DMTS task, the correlation was now closer to zero. A 2 × 2 mixed ANOVA was conducted to better understand the change in correlation of the intercept from the DMTS with experience, with training time point as the within-subject factor (middle vs. end) and age (young vs. old) as the between-subject factor. There was a significant interaction between the intercept and age (*F* (1,16) = 4.99, *p* = .04) and post-hoc tests with a Bonferroni correction showed there was a significant difference in the young group at the middle and end points of training (*t* = -3.7, *p* = .01).

**Table 4:** Spearman correlation matrix between the measures of the cognitive test battery, age, and experience

Spearman’s Correlations - End Point of Training							
Variable		SMTS	RT	DMTS Intercept	DMTS Slope	Reversal Learning	Experience
SMTS	Spearman’s rho	—					
	*p*	—					
RT	Spearman’s rho	0.463 (17)	—				
	*p*	0.061	—				
DMTS Intercept	Spearman’s rho	-0.058 (16)	-0.179 (17)	—			
	*p*	0.832	0.491	—			
DMTS Slope	Spearman’s rho	0.019 (16)	-0.283 (17)	0.358 (18)	—		
	*p*	0.944	0.270	0.144	—		
Reversal Learning	Spearman’s rho	0.195 (18)	0.292 (20)	0.486 (17)	0.338 (17)	—	
	*p*	0.437	0.212	0.048	0.185	—	
Experience	Spearman’s rho	-0.324 (18)	-0.374 (21)	0.022 (18)	0.252 (18)	-0.137 (21)	—
	*p*	0.189	0.095	0.932	0.312	0.554	—
Age	Spearman’s rho	-0.466 (18)	**-0.559 (21)**	-0.022 (18)	0.143 (18)	-0.371 (21)	**0.830 (22)**
	*p*	0.051	**0.008**	0.930	0.571	0.097	**< 0.001**

The number inside the parenthesis is the sample size. Bolded values indicate the result was significant after correcting for multiple comparisons

#### Principal component analysis

Four PCAs were conducted to investigate the cognitive structure at different time points of training for the RT and DMTS tasks. For all tasks, only the dependent measures included in the correlation matrix were used in the PCAs to ensure that all tasks were equally represented and avoid further reductions in power (de Winter et al., 2009; Jensen & Weng, 1994; Mundfrom et al. 2005). Since previous research indicates that the intercept and slope from the DMTS assess different underlying cognitive processes, both were included in the PCAs (White, 2001). Finally, the PCA was based on the Spearman correlation matrix and was unrotated so the first component could account for the maximum amount of variance (Abdi and Williams 2010). By convention, only Eigenvalues larger than 1 were retained.

**Table 5:** The loadings and variance explained for each principal component when assessed at the middle or end point of training for the RT and DMTS tasks, using a Spearman correlation

Principal Component Analysis					
	Middle			End	
	PC1	PC2		PC1	PC2
SMTS	0.00	0.73		0.03	0.77
RT	-0.41	0.79		-0.19	0.88
DMTS intercept	0.86	-0.27		0.82	-0.08
DMTS Slope	0.66	0.30		0.74	-0.19
Reversal Learning	0.68	0.53		0.74	0.47
Eigenvalue	1.8	1.6		1.81	1.62
Variance Explained	0.36	0.32		0.36	0.32

**Fig. 2:**
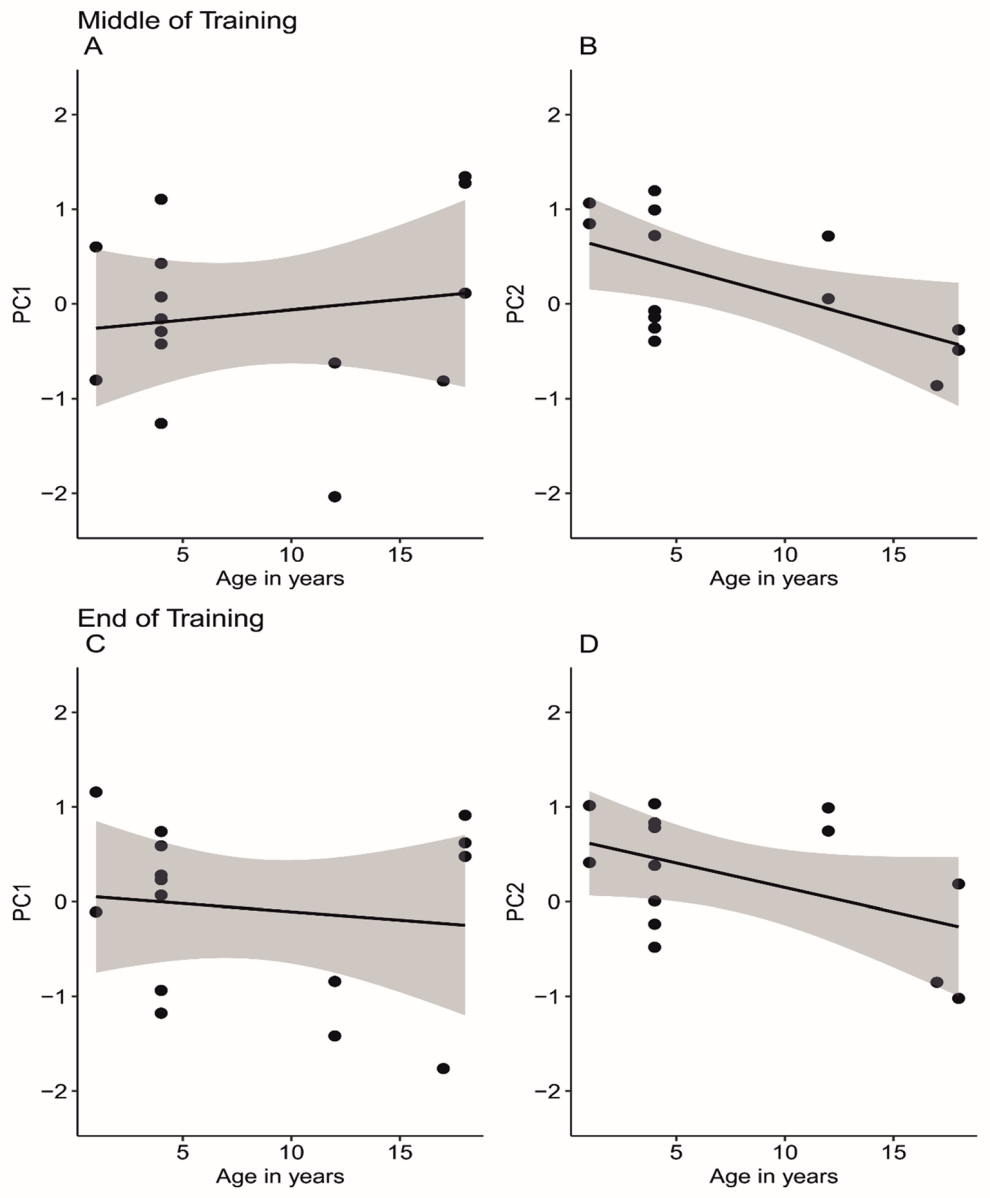
The relationship between subject age and principal components extracted when 5 tasks were included in the PCAs. The top row is from the middle time point of training and the bottom row is from the end point of training for the reaction time and delayed match to sample tasks. Each data point is a subject, the black line is a linear model of the data and the gray shading reflects a 95% confidence interval

For both PCAs, two components with Eigenvalues larger than 1 were extracted (Table 5). When the middle point of training was used for the RT and DMTS task, the loading onto the two components was similar to the pattern in the correlation matrix. Both dependent measures from the DMTS task and the reversal learning task loaded positively onto the first component, while the SMTS did not load onto the first component, and the RT had a negative loading. For the second component, only the intercept for the DMTS task had a negative loading. Both components could explain a similar amount of variance in performance. Similar results were obtained when listwise deletion was used to handle missing data, where only performance from subjects that had completed all tasks in the battery were included (*n* = 15). How the tasks loaded onto the components was similar to the pattern in the correlation matrix, but these tasks were differentially related to age. A Pearson correlation between age and components was performed to better understand this relationship. The correlation between age and PC1 was positive, but close to zero (*r* (15) = 0.15, *p* = .73, Fig. 2a). The correlation between age and PC2 was significantly negative (*r* (15) = − 0.66, *p* = .01, Fig. 2b).

When the end point of training was used for the RT and DMTS task, the task loadings were similar to the middle point of training. The RT and SMTS tasks had negative or weak loadings on the first component and high positive loadings on the second component. The DMTS and reversal learning tasks had a stronger positive loading on the first component. One difference between the middle and end point of training is that the slope for the DMTS had a negative loading on the second component. To understand how age was related to the components at the end time point of training, another Pearson correlation between age and the components was performed. There was a nonsignificant negative correlation between age and PC1 (*r* (15) = -0.13, *p* = .65; Fig. 2c) and a significant negative correlation between age and PC2 (*r* (15) = -0.59, *p* = .02; Fig. 2d).

Due to the potential theoretical issues with including the RT task in the analyses, another set of PCAs were performed with the SMTS, DMTS intercept, DMTS slope, and reversal learning measures. The middle and end time points for the DMTS were analyzed separately. For both time points, two components were extracted and all tasks positively loaded onto the first component (Table 6). Similar to the previous set of PCAs, the SMTS task had a stronger loading on the second component and, at the end point of training, both measures from the DMTS task had a negative loading on the second component.

**Table 6:** The loadings and variance explained for each principal component when assessed at the middle or end point of training for DMTS tasks, using a Spearman correlation

Principal Component Analysis					
	Middle			End	
	PC1	PC2		PC1	PC2
SMTS	0.25	0.9		0.16	0.96
DMTS intercept	0.7	0.51		0.79	-0.26
DMTS Slope	0.72	0.14		0.7	-0.15
Reversal Learning	0.83	0.03		0.81	0.2
Eigenvalue	1.76	1.09		1.8	1.05
Variance Explained	0.44	0.27		0.45	0.26

**Fig. 3:**
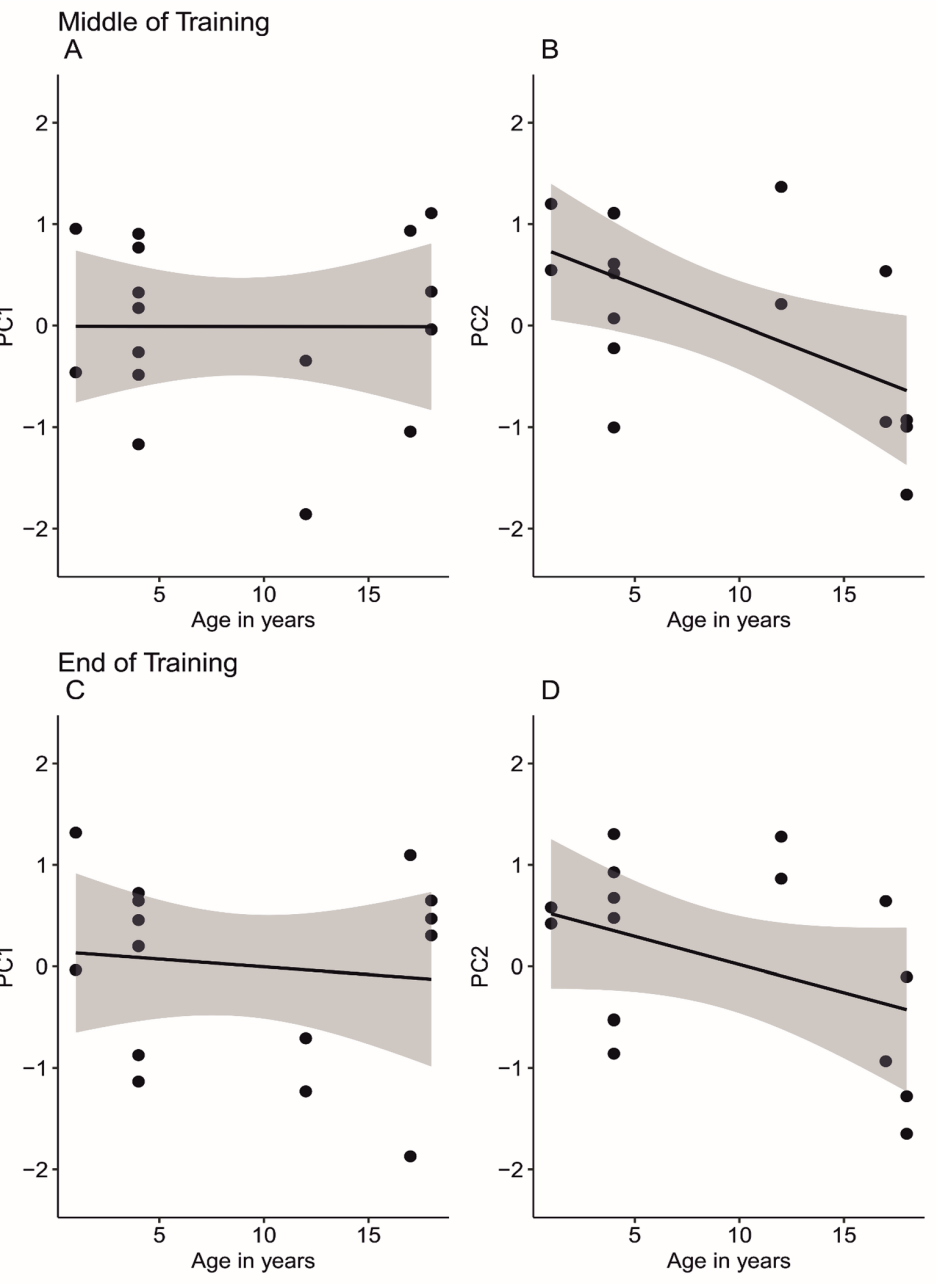
The relationship between subject age and principal components extracted when 4 tasks were included in the PCAs. The top row is from the middle time point of training and the bottom row is from the end point of training for the delayed match to sample tasks. Each data point is a subject, the black line is a linear model of the data and the gray shading reflects a 95% confidence interval

To understand the relationship between age and the components, a series of Pearson correlations were performed. At the middle time point of training, age was not correlated with the first component (*r* (16) = 0, *p* = 1; Fig. 3a), but age was significantly negatively correlated with the second component (*r* (16) = -0.58, *p* = .02; Fig. 3b). At the end point of training, age had a nonsignificant, negative correlation with the first component (*r* (16) = -0.11, *p* = .68; Fig. 3c) and a nonsignificant negative correlation with the second component (*r* (16) = -0.41, *p* = .12; Fig. 3d).

## Discussion

This is the first time that cognitive performance in the pigeon has been systematically investigated using a test battery. The battery was created with the intention of assessing different cognitive domains, including associative learning, cognitive flexibility, STM, and RT. We predicted that performance would positively correlate across tasks. In contrast to our predictions, the correlation matrix was not uniformly positive. The largest correlation among the cognitive tasks was actually negative, between the RT task and the intercept of the DMTS task when assessed at the middle time point of training. In general, the correlation matrix indicated there were two clusters, with the RT and SMTS tasks forming one cluster and the DMTS and reversal learning tasks forming another. Subject age was a potential reason for this clustering since the RT and SMTS tasks were negatively correlated with age, while the intercept for the DMTS task was positively correlated.

When the PCAs included all of the measures, two components were extracted, and the task loadings were similar to what was seen in the correlation matrix. The first component mostly accounted for variance in the DMTS and reversal learning tasks, while the second component accounted for variance in the SMTS and RT tasks. The second component extracted always had a stronger negative correlation with age compared to the first component, further indicating the key difference between these tasks. The RT task specifically seems to be pushing these components in different directions and, as mentioned in the introduction, there are conceptual reasons why the task we designed may not be capturing cognitive performance per se (Jensen 1982; Carlson and Jensen 1982). Additional PCAs without the RT task still extracted two components, but now all of the tasks positively loaded onto the first component extracted. Similar to the previous analyses, the second component had a stronger, negative correlation with age.

The results from the PCAs indicate that there is a factor similar to *g* underlying pigeon performance on cognitive tasks. Even though the RT task had a negative loading, this may have because it was primarily a motor task that was sensitive to age (Godefroy et al. 2010; but see Johnson and Deary 2011). Previous research with other species has similar results, where tasks or measures that have a strong motor component are either weakly related to the other cognitive measures (Shaw et al. 2015) or prevent a *g-like* factor from being found (Locurto et al. 2006). These results indicate that there is a general factor that is present throughout various cognitive tasks across various species (Flaim and Blaisdell 2020; Shaw and Schmelz 2017).

While there is evidence for a general cognitive component, not all tasks had a strong loading. Specifically, the SMTS task had a consistently small loading onto the first component. This could partially be due to the negative effect age had on performance for this task. It could also be due to different stimuli and task demands. The SMTS task used isolated photographs of food or animals against a white background (Blechert et al. 2014), whereas the DMTS and reversal learning tasks used circular stimuli of a solid color. The use of complex photographic stimuli may have provided more alternatives that the pigeons could test (Iwai et al. 1986). Not all of the subjects reached the criterion in the time allotted, and the weak loading could be due to a failure to understand the task requirements. In the DMTS and reversal learning tasks, the task space is more limited, which could make it easier for the subjects to attune to the relevant task demands. Another important difference is that the DMTS and reversal learning tasks could have more interference across trials since the stimuli were used consistently and conditionally associated with reinforcement (Mackintosh et al. 1968; Morand-Ferron et al. 2022; Zentall and Smith 2016). Subjects could have varied in their ability to develop or maintain strategies to protect against interference (Lord et al. 2019). The potentially important role of resistance to interference is similar to results from humans (Burgess et al. 2011) and mice (Matzel & Kolata, 2003).

Of course, the results from this test battery are not a definitive conclusion on the structure of pigeon cognition and there are changes that could be made to strengthen future investigations. The most obvious issue is that the statistical analyses are underpowered (Bonett and Wright 2000; Mundfrom et al. 2005). Future studies should utilize a larger sample size to ensure that the statistical results are robust. Another issue is that our sample has, essentially, a bi-modal distribution of ages. As discussed previously, age seemed to have a strong influence on some of the tasks in the battery and generally it was found that performance decreased with age. This corresponds to more recent research demonstrating that pigeons show similar age-related cognitive declines (Coppola and Bingman 2020; Meier et al. 2021). Unfortunately, age was also confounded with how much previous experience subjects had. Experience could facilitate or hinder new task learning, depending on the compatibility between the task demands and stimulus-reinforcement history. This applies to tasks outside of the test battery and the order in which they experienced the test battery. Determining the impact of experience on performance was not possible with our sample size, thus experience is additional noise in our dataset. It would be beneficial to understand how experience influences performance and it would be interesting to see if young naïve pigeons would yield a similar cognitive structure.

The tasks used in the test battery may have also influenced the results. While there are clear strengths to the tasks used in this battery, there are also weaknesses. Future, stronger test batteries should assess a broader array of cognitive domains. Spatial reasoning in particular would be an excellent addition since there have been investigations directly comparing the abilities of pigeons and humans in a variety of paradigms (Hollard and Delius 1982; Spetch et al. 1996). Non-cognitive factors should also be assessed. since they have been shown to differentially impact performance on seemingly cognitive tasks (Carere and Locurto 2011; Cole and Quinn 2012; Delacoux and Guenther 2023; Isden et al. 2013; Shaw and Schmelz 2017). This could be particularly helpful in understanding why the pattern of correlations changes with task experience (Locurto et al. 2003; Scharfen et al., 2018). Similarly, different reinforcers and apparatuses should be used across tasks. All tasks in the current battery were conducted in a touchscreen operant box for a food reward. It is possible that similarities in performance are due to shared motivational and response requirements. If these components truly reflect an underlying cognitive structure, then they should be replicated across a variety of paradigms. Finally, and on a more practical note, ideally the entire test battery should take far less time to complete. Subjects needed an average of 84.45 days (*SD* = 12.78) to complete the serial reversal learning, SMTS, DMTS, and RT tasks, not including preliminary training. The amount of time to train and test subjects limits the number of tasks that can be included and how feasible it is for other labs to replicate the results. Investigating *g* across a variety of species could help determine if there are consistent neuroanatomical features present in species that exhibit a *g* factor compared to species that do not. Ultimately this test battery is an interesting step towards understanding the general cognitive abilities of the pigeon. Future investigations are sure to yield insights about the structure of general cognitive abilities.

## Data Availability

Support for this research was provided by National Science Foundation grant BCS-1844144 (Aaron P. Blaisdell). Data are available at https://github.com/AaronBlaisdell/IntelligentPigeon.
